# The National Association of Dental Faculties

**Published:** 1904-06

**Authors:** 


### Editorial.

### THE NATIONAL ASSOCIATION OF DENTAL FACULTIES.

It is possible that by the time this reaches our readers the National Association of Dental Faculties will have met at Washington, D. C., and may have concluded its deliberations.

This body has ceased to be a mere instrument to meet annually and to voice the aims and aspirations of the dental colleges. It has become more than this, and it stands to-day as the exponent of the advanced thought of the dental profession of America, and by America is meant not merely the limited boundaries of the United States, but the entire continent. This is written advisedly, for dentistry, as taught since the organization first met has sent its young graduates everywhere, and these represent the thoughts and highest aims of this organization.

If, then, the " Faculties" has become, as represented, the mouthpiece of the profession, it has, as its most important duty, to guard its portals that nothing may enter to mar the confidence heretofore reposed in its decisions.

This body will meet at Washington, D. C., June 9. The writer in a previous number discussed this plan, as at that time proposed, and endeavored to show that no provision was made in the laws governing the organization to warrant any meeting except that provided by the laws heretofore adopted. This, apparently, had no deterring influence, for, upon a vote taken by mail, the majority, it is presumed, decided it was wise to hold this meeting five months after an equally irregular one held at Buffalo in January last, thus making three meetings within ten months for this Association,—those of Asheville, Buffalo, and Washington.

No attempt has been made to disguise the purpose of the two called meetings. It is well known that a certain number of dental colleges are in direct opposition to the course of four years, that went into operation the present session of 1903–04. At the time of the original passage of this act there was but feeble opposition to this measure. It was demanded by the National Association of Dental Examiners and by some of the State boards. Hence opposition, if felt at that time, was silenced.

The meeting at Asheville, N. C., was held preceding the period fixed for beginning this course. The colleges had had no experience as to the results of this advanced time. Harvard refused to comply with the decision of the Faculties, and resigned.

The National Association of Dental Examiners offered a loophole for the weaker dental colleges to escape this, to them, oppressive statute, and then began the efforts to bring about a return to a three years' course. It was attempted at Buffalo and failed, but, not discouraged, the opposition set to work to secure a meeting before the regular period, which would be the last of August at St. Louis. Under the specious pretence that time would not permit careful consideration of other matters at this time, with the National Organization and the Congress to care for, a note was sent round demanding a vote for certain places and for a meeting in June. The result was, it seems, fixed as before stated, at Washington. Who ordered this vote is unknown to the writer, but it is presumed it originated at Buffalo. As this meeting was irregularly called, no authority existed there or elsewhere to call another meeting save and except that provided for under the rules.

The meeting has, therefore, been convened for a special purpose.

What that may be cannot positively be decided at this writing, but presumably it will give an opportunity for those colleges interested in a retrograde movement to push the matter to a finality. The motive which underlies this effort is not difficult to determine. As the course stands at present, it is not to the financial interests of many of the independent dental colleges to continue the extended period. The meeting must be held to meet the time at which announcements are usually issued, and if this retrograde action were adopted it must be entered therein, or a delay of one year would follow. No attempt has been made to disguise this motive by those working in the interest of a three years' course.

The arguments used to sustain this retrograde movement have been previously alluded to and answered, but one used at Buffalo was so intrinsically weak that surprise must generally be felt that men of intelligence could have entertained it as a process of reasoning. This was that three years of nine months would be practically equal to four years of seven months, the first making a college course of twenty-seven months and the latter one of twenty-eight. It seems to have been forgotten that the most strenuous advocates of the four years have been the departments of universities, and these have had, for a number of years, courses of nine months each. The results have not been satisfactory, and they have insisted on an extension of time. That the schools not equally as well fitted for training undergraduates should be able to accomplish what these have found beyond their power must impress itself upon every logical mind.

At this writing, a month in advance of the meeting, it is impossible to foretell with any accuracy what may result from this meeting, but it is earnestly desired that wise counsels may prevail and that the standard will be left as it is at present.

Most of the dental colleges of this country are organized upon a weak financial foundation. The majority are comparatively new and are dependent upon the income from students, and it is natural that the faculties view the prospect with serious misgivings. It means, from their view, a dissolution of the faculties and serious financial loss. They will therefore, be unable to regard the ethical side of the question at Washington, and if able to force their opinions it may be that their retrograde ideas may prevail and a three years' course be ordered.

The object of this article is not to influence legislation on this subject, for it will be too late even to be read by those most interested. Its motive is to give the world at large an insight into the influences that have led up to this effort. If this be successful, those interested in advanced dental education should understand the influences leading to such a disastrous ending of twenty years of earnest work.

It is a mistake to suppose that this first year of the four years' course represents the future. It will, of course, require some years to accustom the minds of parents and guardians to the new order of things, but experience has demonstrated that financially the schools are, in the end, equally as well off with the higher standard as they were with the lower. Every advance in dental teaching has been followed by a period of depression, to be again followed by an enlarged matriculation list, and this means more than mere financial benefit, for it brings with it a higher class of men and an equally higher standard of character.

The writer has, however, very little interest in the commercial side of dental education. The aim of the active workers has been, from the time of Harris and Hayden, to carry dental training to its utmost limits, and if this means the destruction of means and methods that may have become antiquated or insufficient, the earlier this is accomplished the better it will be not only for our profession, but for the world at large.

